# Scoping review of the role of pharmacometrics in model-informed drug development

**DOI:** 10.1007/s10928-025-10005-8

**Published:** 2025-10-15

**Authors:** Amruta Gajanan Bhat, Euibeom Shin, Amit Roy, Murali Ramanathan

**Affiliations:** 1 Artificial Intelligence and Clinical Pharmacology Laboratory, Department of Pharmaceutical Sciences, University at Buffalo, The State University of New York, 355 Pharmacy, Buffalo, NY 14214-8033 USA; 2 PumasAI, Dover, DE USA

**Keywords:** MIDD, Pharmacometrics, AI, Study design

## Abstract

Model-informed drug development (MIDD) is a framework that utilizes quantitative modeling and simulation methods to integrate nonclinical and clinical data, as well as prior knowledge, to inform drug development and regulatory decision-making. This scoping review examines the current MIDD landscape with a focus on pharmacometrics. It will clarify essential MIDD concepts and provide a taxonomy of terms. Additionally, it will evaluate the implications of the International Council for Harmonisation (ICH) M15 MIDD draft guidelines and summarize case studies that illustrate the use of MIDD in various drug development and lifecycle management scenarios. The diverse case studies span various therapeutic areas, small molecules, monoclonal antibodies, and long-acting injectable dosage forms. The ICH M15 MIDD guidelines aim to align the expectations of regulators and sponsors, support consistent regulatory decisions, and minimize errors in the acceptance of modeling and simulation. MIDD is a transformative and rigorous process that fosters collaboration between industry and regulatory agencies during drug development.MIDD has enabled accelerated approvals of drugs for pediatric conditions and rare diseases, where it can be difficult to recruit enough patients for efficacy studies. MIDD has contributed to the clinical pharmacology strategies that have successfully allowed dose extrapolation to related disease indications, dosage forms, and clinical populations without additional clinical trials.

**Supplementary Information:**

The online version contains supplementary material available at 10.1007/s10928-025-10005-8.

## Introduction

Model-informed drug development (MIDD) is a framework that leverages quantitative models based on preclinical and clinical data to inform drug development decisions [1–3]. The International Council for Harmonisation of Technical Requirements for Pharmaceuticals for Human Use (ICH), which connects regulatory agencies and pharmaceutical industry representatives to build consensus on technical standards for pharmaceutical products, issued the ICH M15 draft guidelines for MIDD in November 2024 [4].

In this review, we (i) clarify key MIDD concepts and provide a taxonomy of terms, (ii) assess the landscape for pharmacometrics in MIDD, including the implications of the ICH M15 draft guidelines, and (iii) summarize representative real-world case studies illustrating the utilization of MIDD in drug development and drug lifecycle management.

The scoping review framework was selected because it can map key concepts, definitions, and conceptual boundaries for a broadly impactful and potentially transformative framework, such as MIDD [5, 6]. This review can also serve as a tutorial for scientists new to drug development and MIDD processes, as well as provide experienced scientists with a perspective on diverse MIDD successes and the ICH M15 guidelines.

Pharmacokinetics (PK), the time course of drug concentrations, is often summarized in the acronym ADME, which refers to the processes of drug absorption, distribution, metabolism, and elimination [7]. Each of these underlying processes can have multiple biochemical and physiological determinants, with subversive interactions that are drug, disease state, and patient-specific, contributing to the variability of drug concentrations. Pharmacodynamics (PD), which refers to the dynamics of drug responses, exhibits greater variability due to the convolution of PK variability with variations in pharmacological signaling and response mechanisms. Population PK-PD (PopPK-PD) modeling [8, 9], which typically leverages nonlinear mixed-effects modeling of compartmental PK and PD models, has emerged as a preeminent methodology for dose-exposure-response (E-R) predictions in MIDD. It is widely used to characterize drug concentrations and variability in effects, as well as to perform clinical trial simulations [10, 11]. The most frequent (~ 70%) application of physiologically based PK (PB-PK) models in drug development and regulatory settings remains the prediction of drug-drug interactions with enzymes and transporters [12].

The utilization of modeling and simulation methods can lead to greater efficiency in drug development and regulatory evaluation processes, as it can address a broader range of dose-exposure responses, product design, special populations, and disease-related questions. MIDD is a useful framework for generating evidence with modeling and simulation in support of strategically planned regulatory interactions for drug development.

## Regulatory history and evolution of MIDD

MIDD emerged as regulatory agencies, including the United States Food and Drug Administration (FDA) and European Medicines Agency (EMA), recognized that it is impractical to address all drug development and regulatory questions based solely on the outcomes of well-controlled Phase 3 clinical studies, which rely on statistically guided decision-making from clinical efficacy and safety outcome endpoints [13, 14]. Exemplars include the development of drugs for rare diseases and pediatric conditions [15]. In 2019, the FDA issued a draft guidance describing situations in which the conventional substantial evidence regulatory standard for approval (two adequate and well-controlled Phase 3 studies or, alternatively, a single trial plus confirmatory evidence after implementation of the FDA Modernization Act) could be appropriate [16]. The EMA also provides guidance on this issue [14]. These approaches could be complemented by modeling and simulation methods, which integrate existing knowledge and information collected during the drug development program.

The evolution of regulatory processes underlying MIDD began in the mid-1990s. The FDA issued a PopPK guidance for industry in 1999 [11, 17] and a guidance for study design, data analysis, and regulatory applications of E-R relationships in 2003 [18]. The FDA’s Division of Pharmacometrics (DPM) issued a 10-year strategic plan in 2010 [19, 20].

The evolution of the MIDD framework can be traced to several preceding efforts, including model-based drug development (MBDD) [21] and model-informed drug discovery and development (MID3) [22]. In 2017, best practices for MID3, defined as a “*quantitative framework for prediction and extrapolation*,* centered on knowledge and inference generated from integrated models of compound*,* mechanism*,* and disease level data*,* aimed at improving the quality*,* efficiency*,* and cost-effectiveness of decision-making*” were published by the European Federation of Pharmaceutical Industries and Associations (EFPIA) MID3 workgroup following a EMA workshop [23]. MID3 envisioned a role for modeling and simulation in early drug discovery, preclinical research, and throughout the entire drug lifecycle, to enhance drug development and regulatory processes. The concept of utilizing risk-based assessment, which emerged from this effort, was adopted in the ICH M15 guidelines.

The Prescription Drug User Fee Act Reauthorization VI (PDUFA) of 2017 catalyzed efforts by the FDA to incorporate biomarkers, real-world evidence, and alternative clinical trial designs into the drug approval process [24, 25].

The extent to which MIDD is integrated into regulatory decision-making can vary across agencies, between applications, and even within the same agency for similar submissions, which provided an impetus for harmonization. The experience with the FDA MIDD pilot program spurred the Pharmaceutical Research and Manufacturers of America (PhRMA) trade group to engage with the ICH on the need for a global guideline. As a result, in November 2021, the ICH Assembly endorsed the formation of a working group to develop a concept paper on General Considerations for Model-Informed Drug Development (MIDD). The draft M15 Guideline on “General Principles for Model-Informed Drug Development (MIDD)” was endorsed by the ICH Assembly at its meeting in Montreal, Canada, and released for public consultation in November 2024 [26] with the statement, “*The overarching Guideline is expected to cover the general principles and good practices for the use of MIDD and will harmonise expectations regarding documentation standards*,* model development*,* data used in the analysis*,* model assessment*,* and its applications*.” The FDA released the ICH M15 guidelines for public comment shortly thereafter [27]. The timelines for adopting and implementing the ICH M15 guidelines are the end of 2025 and 2026.

The ICH M15 MIDD draft guidelines aim to align the expectations of regulators and sponsors, support consistent regulatory decisions, and minimize errors in the acceptance of modeling and simulation to inform drug labels. The ICH M15 MIDD framework is based on the American Society of Mechanical Engineers ASME 40-2018 standard [28] for evaluating the relevance and adequacy of verification and validation (V&V) activities to ensure the credibility of computational models. Kuemmel et al. proposed applying the credibility framework to MIDD to standardize model assessments independently of the modeling approach [29]. They illustrated the principles of credibility assessment for a PB-PK model [29]. In 2023, the FDA adopted these concepts and published guidelines [30] that offer a more general framework for demonstrating the credibility of computational modeling and simulation in medical device regulatory submissions. These guidelines incorporate various categories of credibility evidence.

## MIDD and taxonomy of MIDD terms

**Fig. 1:**
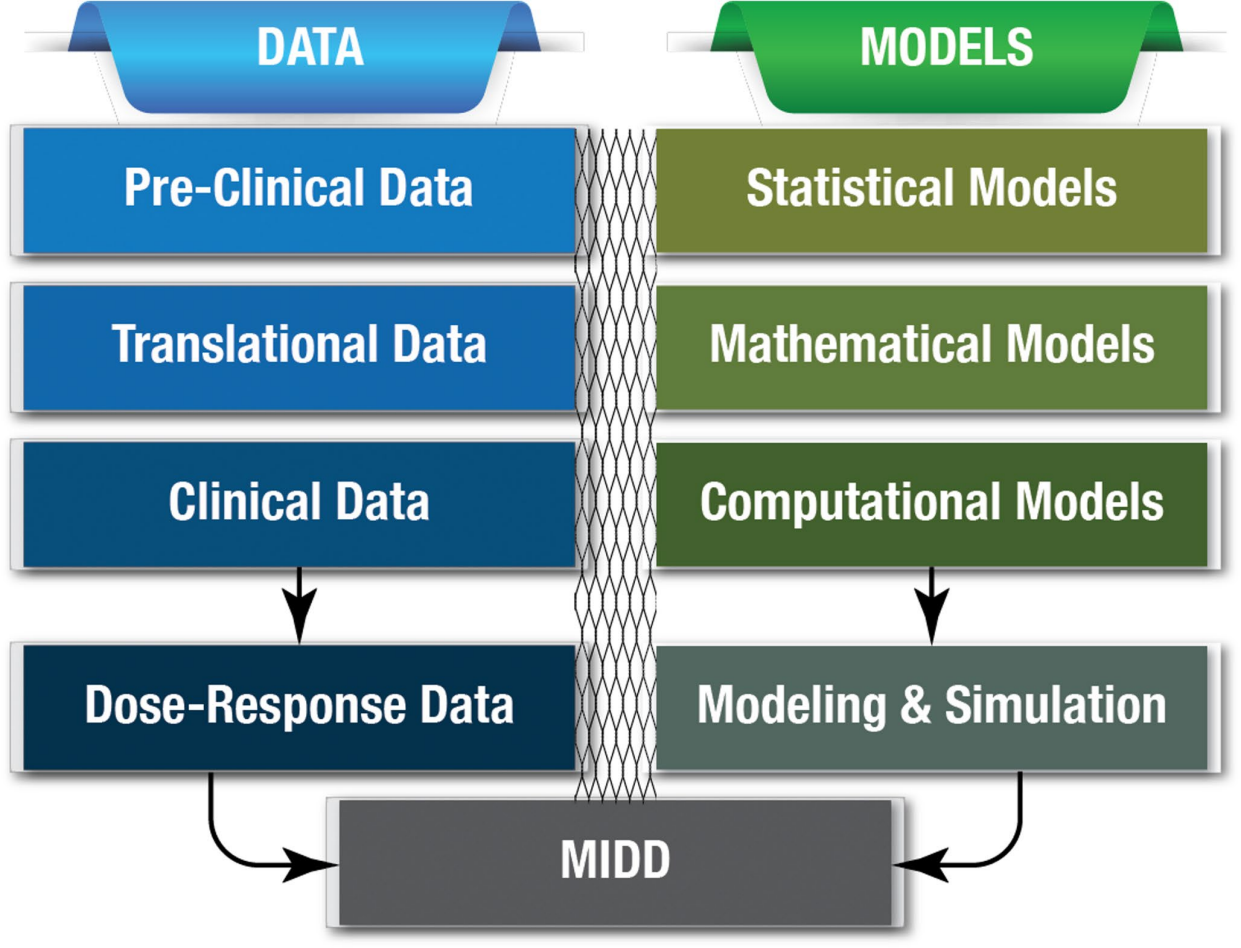
Schematic highlighting how exposure-response from pre-clinical, translational, and clinical experiments can be analyzed with mathematical, statistical, and computational modeling and simulation tools in model-informed drug development (MIDD)

**Fig. 2:**
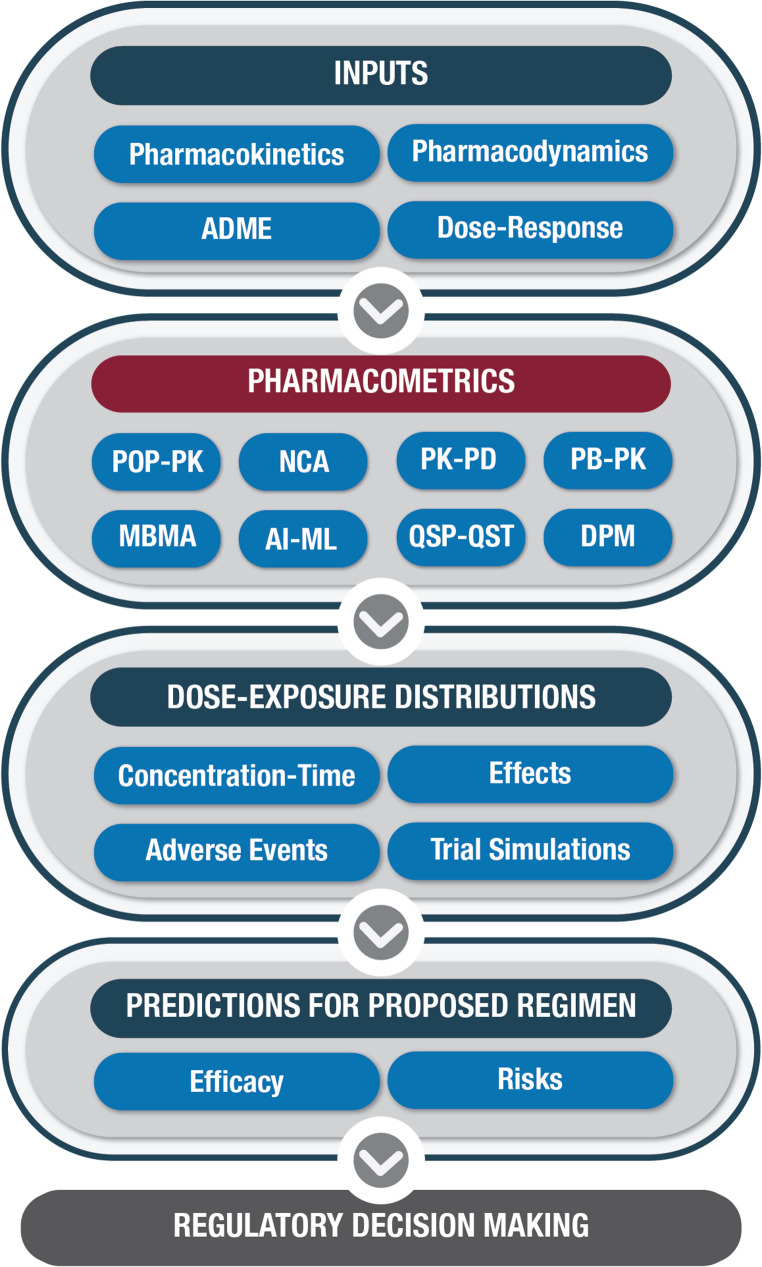
Schematic the inputs to pharmacometric methods and the resulting modeling output. Abbreviations: ADME: Absorption, distribution, metabolism, and elimination; Pop-PK: population pharmacokinetics; NCA: non-compartmental analysis; PK-PD: pharmacokinetic and pharmacodynamic modeling; PB-PK: physiologically based pharmacokinetic modeling; MBMA: model-based meta-analysis; QSP: quantitative systems pharmacology; QST: quantitative systems toxicology; ML: machine learning; AI: artificial intelligence; DPM: Disease progression modeling

MIDD can be viewed as both a technical framework and a regulatory tool. As a technical framework, it facilitates the application of a wide range of quantitative models, including mathematical, statistical, and computational models, by pharmaceutical scientists to support drug development and informed decision-making (Fig. 1). As a regulatory tool, it promotes early interaction between drug developers and regulatory agencies to discuss how modeling could be used to address drug development questions of interest for regulatory decision-making (Fig. 1 , 2).

### Definition of MIDD

In the ICH M15 draft guideline [4], “*MIDD is defined as the strategic use of computational modeling and simulation (M&S) methods that integrate nonclinical and clinical data*,* prior information*,* and knowledge (e.g.*,* drug and disease characteristics) to generate evidence*.” The pharmacometrics methods explicitly included in the ICH MIDD framework include PopPK, PB-PK, biopharmaceutics, dose-E-R analysis, model-based meta-analysis, quantitative systems pharmacology and toxicology, agent-based models, and disease progression models. Notably, the guidance also broadly encompasses AI/ML methods [4].

### MIDD process

The main objectives of the ICH M15 guidelines development process were to provide recommendations that enable (i) structured planning, development, and documentation of modeling activities, (ii) harmonized assessment process for MIDD evidence, and (iii) alignment and consistent communication across the various drug development disciplines by providing a well-defined taxonomy of terms and terminology.

An important efficiency-enabling aspect of MIDD is the structured consultative framework that fosters early alignment and common expectations between the drug sponsor and regulatory agency.

### Taxonomy of MIDD terms

**Fig. 3:**
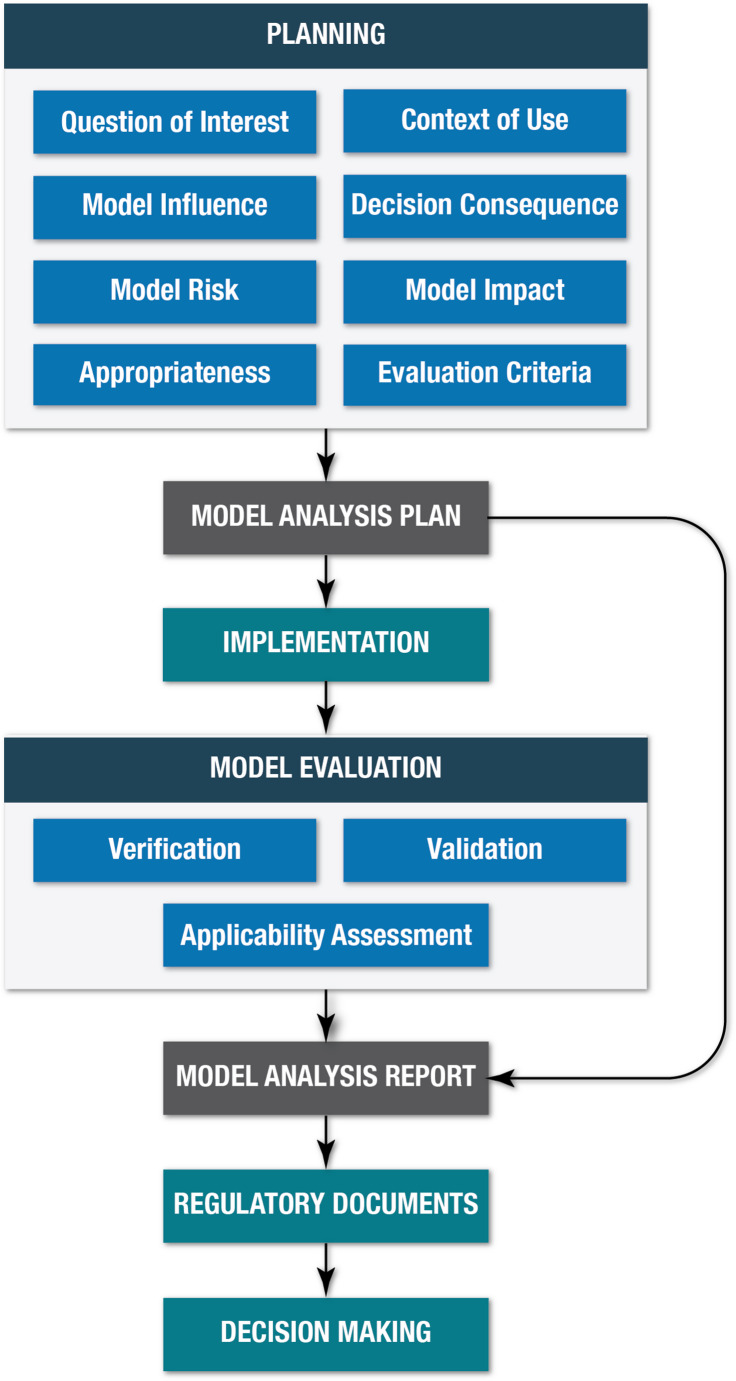
Overview of the stages of MIDD, as outlined in the ICH M15 guideline, includes Planning and Regulatory Interaction, Implementation, Evaluation, and Submission. Abbreviations: Planning: Planning and Regulatory Interaction; Decision Consequence: Consequence of Wrong Decision; Appropriateness: appropriateness of proposed MIDD; Evaluation Criteria: technical criteria for model evaluation and model outcomes; Regulatory Documents: Documentation for Regulatory Interactions and Submissions

The stages of MIDD activities include Planning and Regulatory Interaction, Implementation, Evaluation, and Submission (Fig. 3). The ICH M15 draft guidelines identify, define, and operationalize a useful taxonomy of MIDD-related terms that can be confusing to those unfamiliar with MIDD [4].

The definitions are from the ICH M15 guidelines [4]. The ICH copyright is acknowledgedStage 1 begins with planning that defines the Question of Interest (QOI), Context of Use (COU), Model Influence, Decision Consequences, Model Risk, Model Impact, Appropriateness, and Technical Criteria. Interaction with regulatory agencies during this stage aims to inform decision-making, verify the appropriateness of the proposed MIDD approach, and establish technical criteria for model evaluation and model outcomes. All these steps are documented in a Model Analysis Plan (MAP). The MAP document typically consists of Introduction, Objectives, Data, and Methods sections. These terms are explained in Table 1.

**Table 1:** Taxonomy of MIDD terms for the model analysis plan and model evaluation

Key assessment elements
Question of Interest: The question that MIDD is intended to answer. State the Question of Interest. Context of Use: A description of the model(s) and its specific role and scope to answer the Question of Interest. Provide a concise, clear, and explicit description of the model, the data used to build the model, the specific role of the model outcomes, and the other data or evidence that will contribute to the answer to the Question of Interest. Model Influence: The intended weight of the model outcomes in decision-making considering the contribution of other relevant information. Describe the Model Influence; rate it as low, medium, or high considering other relevant information (e.g., nonclinical and clinical) to inform decision-making; and justify the rating. Consequence of Wrong Decision: The consequences (e.g., with respect to patient safety and/or efficacy) if a wrong decision is made, based on all available information. Describe the consequence of a wrong decision; rate it as low, medium, or high based on the severity of the consequences a wrong decision may have on patient safety and efficacy; and justify the rating. Model Risk: The contribution of the model outcomes to a possible wrong decision and subsequent potential undesirable consequences. Describe the risk; rate it as low, medium, or high based on the Model Influence rate and the Consequence of a Wrong Decision rate; and justify the rating. Model Impact: The contribution of the model outcomes in relation to current regulatory expectations or standards in answering the Question of Interest. Describe the impact; rate it as low, medium, or high considering current regulatory expectations or standards; and justify the rating.
MIDD planning stage
Appropriateness of Proposed MIDD: The rationale for why the proposed MIDD is suitable to answer the Question of Interest and cover the related key assumptions and required data. Include a description and justification sufficient to facilitate regulatory interaction on the appropriateness of the proposed MIDD to answer the Question of Interest. Technical Criteria: A summary and rationale of the key criteria for Model Evaluation and model outcomes to establish the acceptability of the model (e.g., using an acceptance standard such as bioequivalence acceptance limits). Include a description of the Technical Criteria for the assessment of Model Evaluation and model outcome. This should include sufficient details on the relevant metric(s).
MIDD evidence submission stage
Model Evaluation: A brief discussion of the key results and conclusions of the technical evaluation4 of the model. Describe the key results and how they compare to and fulfill the Technical Criteria and conclude on the acceptability of the model performance and model outcome, with details being provided in the appropriate regulatory documentation. Outcome of the MIDD Evidence Assessment: A concise summary of the multidisciplinary assessment of the MIDD evidence to answer the Question of Interest. Provide a multidisciplinary integrative assessment and conclusion for the acceptability of the MIDD evidence to contribute to the answer to the Question of Interest, referring to the MIDD assessment framework elements.

The definitions are from the ICH M15 guidelines [4]. The ICH copyright is acknowledged

In Stage 2, the model is implemented and evaluated. Model Evaluation involves Verification, Validation and Applicability steps to ensure credibility and rigorously interpret the results from implementing the MAP (see Table 1 for definitions, which are recapitulated as is from the ICH M15 draft guidelines to avoid confusion).

At the end of Stage 2, a Model Analysis Report (MAR) summarizing the modeling results is submitted to regulators. It should include details on data selection, inclusion/exclusion criteria, data transformations, and imputation performed on the data. The MAR includes the following sections: executive summary, introduction, objectives, data and methods, results, discussion, conclusion, references, and appendices.

In the Submission stage, all necessary documents, including the MAP, MAR, and supporting references, are compiled and submitted to regulatory agencies. This documentation informs regulatory decision-making and facilitates the acceptance of the MIDD approach.

## Current landscape for pharmacometrics in MIDD

**Fig. 4:**
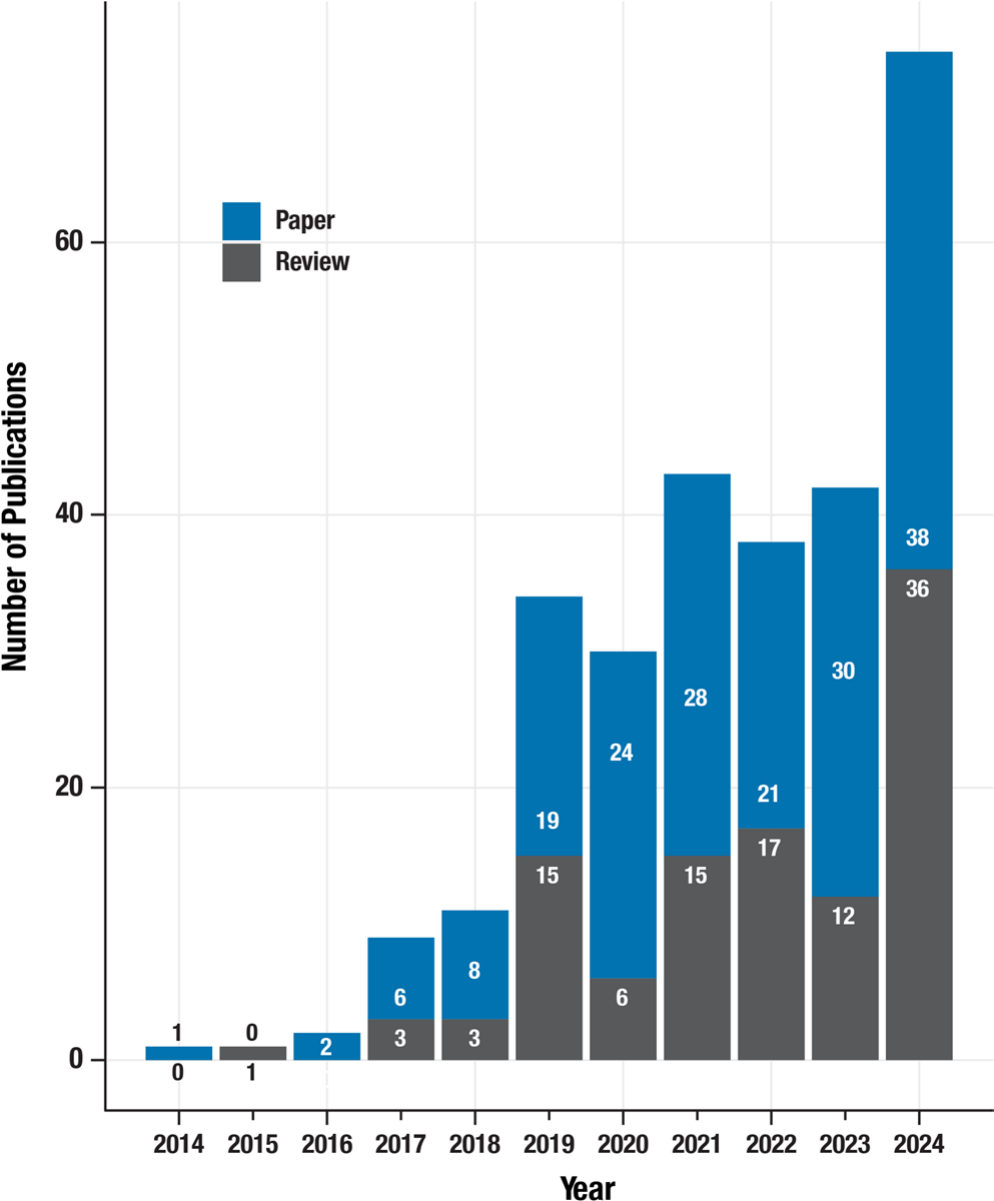
The number of MIDD publications was categorized by review (gray bars) and non-review (Paper, blue bars) publication types between 2014 and 2024. The number of publications is shown in the bars

A search of the PubMed bibliographic database with the search term “Model-informed“[All Fields] AND “drug development“[MeSH Terms] OR “MIDD“[All Fields] NOT “Maternal diabetes” [All Fields] NOT “maternally inherited diabetes and deafness” [All Fields] NOT “monoclonal immunoglobulin deposition disease“[All Fields] was conducted on January 11, 2025. The results were downloaded and manually reviewed to delete references extraneous to MIDD. The process yielded 181 (non-review) papers and 110 reviews for the period 2014–2025. Figure 4 shows the number of publications categorized by review and non-review publication types between 2014 and 2024. The number of publications has continued to increase rapidly since 2019. However, this is an incomplete representation of the MIDD work done prior to the term being coined.

**Fig. 5:**
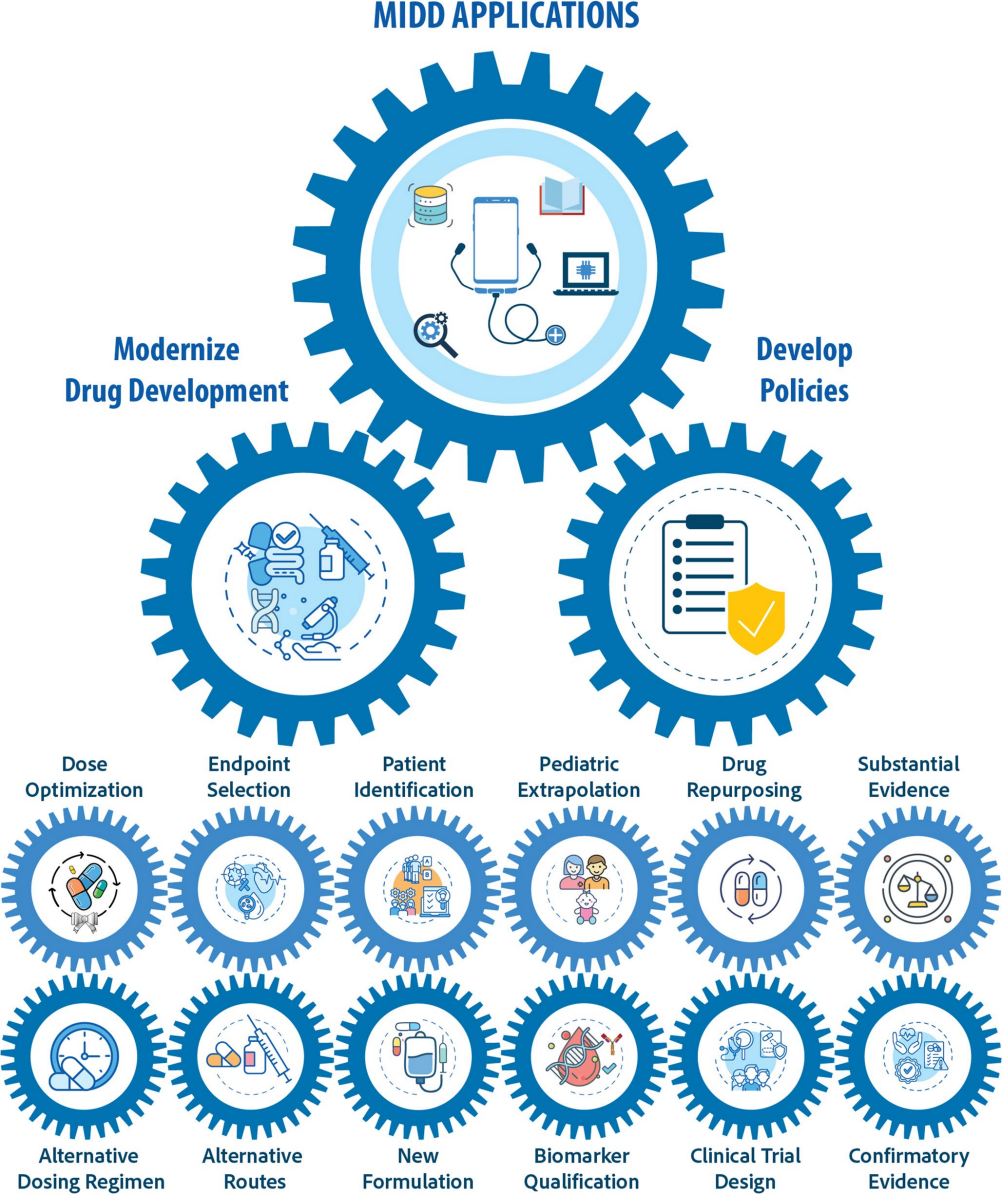
Schematic of the diverse applications of MIDD

MIDD is rapidly becoming an important tool for modernizing and streamlining the drug development process and developing regulatory guidelines. Figure 5 shows some of the diverse development applications in which MIDD can be used.

## Case studies

The principle that drug efficacy and safety are driven by exposure is the underlying reason that MIDD brings efficiencies compared to conventional statistical methods, which do not leverage knowledge of the mechanism of action and E-R relationships of a drug.

**Fig. 6:**
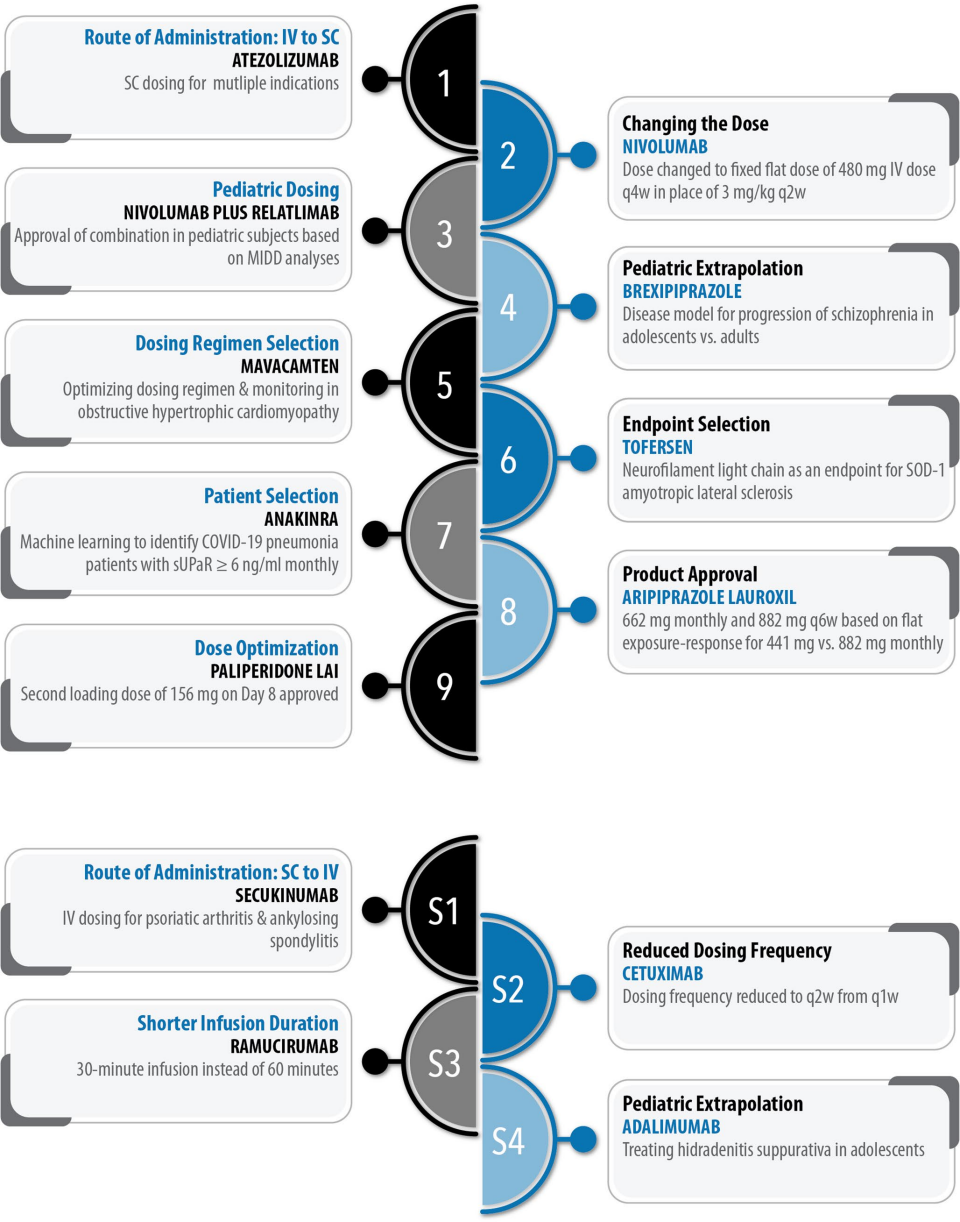
A bulleted summary of the case studies of the different applications of MIDD. The goals of the MIDD, the target drug, and the outcome are summarized. Abbreviations: IV: intravenous; LAI: long-acting injectable; SOD-1: superoxide dismutase-1; sUPaR: soluble urokinase plasminogen activator receptor. Case studies S1-S4 are summarized in Supplementary File

The case studies highlighted in Fig. 6 demonstrate the diverse range of drugs for which MIDD has been utilized to address distinct clinical needs in drug development and regulatory evaluation. Four additional case studies are summarized in the Supplementary File. These case studies employed a robust understanding of the drug’s mechanism of action and pharmacometrics.

## Case study 1: change of route of administration from intravenous to subcutaneous for atezolizumab [31]

Historically, randomized controlled efficacy studies have been required to obtain approval of subcutaneous (SC) formulations of biologics initially approved for intravenous (IV) administration [32]. A major shift in the regulatory strategy to bridge IV to SC formulations was ushered by the clinical development program for SC rituximab, which was successful in establishing non-inferiority of observed trough concentation $\left(C_{trough}\right)$ as the primary endpoint of the pivotal Phase 3 study, supported by efficacy as a secondary endpoint. The acceptance of $\:{C}_{trough}$ as the basis of PK non-inferiority was based on establishing this measure of efficacy as the driver of efficacy. The FDA and other regulatory agencies have required demonstrating PK non-inferiority of time-averaged concentration $\left(C_{avg}\right)\:$ for drugs for which $\:{C}_{trough}$ is not established as a driver of efficacy [33, 34]. Demonstrating PK non-inferiority of $\:{C}_{avg}$ requires calculations of the area-under-the-curve $\left(AUC\right)\:$ of PK profiles, which are challenging to obtain in patients, using non-compartmental (NCA) analysis. A more feasible approach to determining $\:AUC$ in patients is by PopPK analysis of sparsely sampled data. The PopPK analysis approach was utilized to obtain the regulatory approval of SC atezolizumab based on co-primary endpoints $\:{C}_{trough}$ and $\:AUC$ after the first dose [35].

### Atezolizumab

Atezolizumab (TECENTRIQ^®^) is a humanized monoclonal IgG1 antibody developed by Genentech, targeting programmed death-ligand 1. The IV infusion dosing regimen of 1200 mg every 3 weeks, 840 mg every 2 weeks, and 1680 mg every 4 weeks of atezolizumab was approved in the United States, Europe, and elsewhere to treat locally advanced or metastatic non-small cell lung cancer (NSCLC), urothelial carcinoma (UC), locally advanced or metastatic triple-negative breast cancer (TNBC), and extensive small-cell lung cancer (SCLC).

Based on patient preferences for convenience, less pain, and shorter clinic visits, along with its potential to help clinicians streamline workflows, reduce drug waste, and minimize IV-related adverse events, Genentech sought approval for subcutaneous administration instead of IV to reduce healthcare costs and improve resource efficiency.

### QOI

Can an SC dose match IV exposure, maintain tolerability, and enable noninferior PK?

### Atezolizumab pharmacometric model

The study employed both non-compartmental analysis and a PopPK model. Parameters (bioavailability and absorption rate) were added to an existing IV popPK model to describe SC absorption profiles. Exploratory graphical analysis was also used to observe the impact of the site of administration.

Cycle 1 $\:{C}_{trough}$ and $\:AUC$ from SC 1800 mg in the thigh showed higher exposure. The PopPK simulations suggested that exposure after 1875 mg SC was close to the observed 1800 mg dose. The safety of SC atezolizumab was consistent with the known risks of atezolizumab IV, with no new safety signals observed. This combination of clinical and simulated data supported the regulatory approval of SC atezolizumab at a dose of 1875 mg every 3 weeks, administered in the thigh [31].

Notably, although the pivotal IV to SC bridging study was conducted in NSCLC patients, the model-based bridging enabled the approval of SC (TECENTRIQ HYBREZA™) across multiple indications [36].

## Case study 2: reducing the dosing frequency for nivolumab [37]

Nivolumab (OPDIVO^®^), developed by Bristol-Myers Squibb, is a fully human IgG4 monoclonal antibody that targets programmed death-1 (PD-1). Nivolumab at 3 mg/kg administered via IV infusion over 60 min once every 2 weeks, was initially approved by the FDA in 2014 for the treatment of metastatic melanoma, and subsequently for NSCLC, renal cell carcinoma (RCC), UC, classical Hodgkin’s lymphoma (cHL), head and neck squamous cell carcinoma (SCCHN), and hepatocellular carcinoma (HCC) [38].

Bristol-Myers Squibb sought approval for two new dosing regimens: a flat IV 240 mg Q2W regimen and a regimen with a lower dosing frequency of 480 mg IV every 4 weeks (Q4W), to provide healthcare providers and patients with additional flexibility. The proposed 480 mg Q4W regimen was supported by PK modeling, E-R analyses, and safety data without direct clinical efficacy data.

### QOI

What is the alternative dose of nivolumab for treating different tumors?

Subsequently, MIDD was utilized to change the approved dosage regimen to IV 240 mg Q2W, and to introduce an optional posology of IV 480 mg Q4W. The change to a fixed flat dose of 240 mg in place of 3 mg/kg was motivated by a desire to make it more convenient to prepare the dose in the clinic, and to mitigate the risk of dosing errors [39], and the introduction of the less frequent Q4W dosing schedule was motivated by a desire to reduce the burden of clinic visits on patients and caregivers [40]. The fortuitous approval of the Q4W dosing frequency of nivolumab before the COVID-19 pandemic enabled cancer patients being treated with nivolumab to avoid unnecessary exposure to infection by reducing the frequency of clinic visits. The success of the nivolumab MIDD posology bridging strategy led to the release of FDA and EMA Guidances on PK criteria that are adequate to support the approval of alternative dosing regimens for other anti-PD-1/anti-PD-L1 drugs [41, 42].

### Nivolumab pharmacometric model

The nivolumab Q2W to Q4W MIDD bridging strategy was enabled by robust dose-E-R data that covered a 10-fold dose range (1 mg/kg Q2W to 10 mg/kg Q2W) [43]. These robust data facilitated the development of PopPK and E-R models of efficacy and safety, which demonstrated similar efficacy and safety for advanced cancer indications: melanoma, RCC, and NSCLC [44]. The nivolumab PopPK model was also applied to predict and compare exposures for Q2W and Q4W dosage regimens in other indications for which nivolumab Q2W was approved.

A PopPK model was developed using data from 4166 patients across various tumor types, with dosing regimens ranging from 0.1 to 10 mg/kg Q2W and 0.3 to 10 mg/kg Q3W, to predict exposure for the 480 mg Q4W dosing regimen. As no PK data were available for nivolumab 480 mg Q4W, the predicted exposures were compared to the 3 mg/kg Q2W and 240 mg Q2W regimens.

E-R analyses showed that 480 mg Q4W achieved comparable efficacy to 3 mg/kg Q2W and 240 mg Q2W. Safety evaluations indicated similar adverse event profiles across all dosing regimens.

The PopPK analyses provided exposure and safety bridging to support the benefit-risk assessment of the 480 mg Q4W nivolumab dosing regimen without requiring clinical trials. This dosing regimen obtained regulatory approval for eight tumor indications [38].

## Case study 3: pediatric dosing of nivolumab plus relatlimab [45]

The criteria for PK-based extrapolation of adult efficacy to pediatric patients were first established by the pediatric extrapolation decision tree published in the FDA Guidance on Exposure-Response Analyses [46]. Subsequently, the EMA adopted this approach, which led to the ICH E11A Guidance [47].

The two key criteria required for PK-based extrapolation from adults to pediatric patients are similarity of disease and exposure. PK-based extrapolation requires establishing a robust PopPK model in adults and pediatric patients to recommend pediatric dosage regimens that achieve similar exposures in adults and pediatric patients.

The approval of pediatric indications almost always lags that of adult indications, as the pediatric PK data are generally unavailable when the approval for the adult indication is obtained. An exception is the initial approval of the nivolumab plus relatlimab combination (OPDUALAG^®^) for both adult and adolescent patients with advanced melanoma [48].

Relatlimab, developed by Bristol Myers Squibb, is a monoclonal antibody that targets the LAG-3 protein on immune cells. The combination of nivolumab and relalimab was approved by the FDA in 2022 for the treatment of unresectable or metastatic melanoma in adults and pediatric patients aged 12 years or older.

The combination of nivolumab and relatlimab had demonstrated a tolerable safety profile in patients with previously treated melanoma. To evaluate its safety and efficacy in previously untreated metastatic or unresectable melanoma, this combination of 160 mg of relatlimab and 480 mg of nivolumab, administered as a single 60-minute IV infusion every 4 weeks, was further investigated.

### QOI

Does the fixed-dose combination regimen of nivolumab 480 mg plus relatlimab 160 mg administered every 4 weeks result in comparable PK, safety, and efficacy to the individual dosing in both adults and pediatric (≥ 12 years, ≥ 30 kg) patients?

### Nivolumab plus relatlimab pharmacometric model

Relatlimab PK was modeled using a 2-compartment, zero-order IV infusion model with parallel linear and nonlinear clearance (*n* = 1,713). Nivolumab PK, which included pediatric data for extrapolation, was modeled using a linear 2-compartment model with zero-order IV infusion, first-order elimination, and time-varying clearance (*n* = 2,974). Models were developed in stages and evaluated using standard goodness-of-fit plots, prediction-corrected visual predictive checks, and first-order conditional estimation (FOCEI) in NONMEM.

A virtual adolescent population was generated using NHANES data to simulate body weight, age, sex, and race distributions for exposure predictions. These simulated data were used to extrapolate pediatric dosing using PopPK models to predict adolescent exposures (≥ 12 years, ≥ 30 kg) based on adult PK data. Clearance and volume obtained from pediatric data from the nivolumab study were applied to relatlimab. Predicted adolescent exposures aligned with adult exposure ranges, supporting a bridging strategy without requiring clinical pediatric data.

The simultaneous approval of the nivolumab plus relatlimab combination (OPDUALAG^®^) for both adult and pediatric indications was enabled by the MIDD bridging of a robust PopPK model for nivolumab in adults and pediatric patients [49], and a PopPK model of relatlimab in combination with nivolumab [50]. The availability of these models enabled dose recommendations for pediatric patients. The totality of the MIDD evidence led to the simultaneous regulatory approval of the nivolumab plus relatlimab fixed-dose for adult and pediatric indications in both the US and EU without the need for clinical trials in pediatric patients [50, 51].

## Case study 4: pediatric extrapolation for brexpiprazole [52]

Brexpiprazole (Trade Name: REXULTI^®^), an atypical antipsychotic co-developed by Otsuka Pharmaceutical and H. Lundbeck A/S, was approved by the FDA in 2015 for the adjunctive treatment of major depressive disorder and schizophrenia [53].

The FDA recommended starting dose of brexpiprazole for schizophrenia in pediatric patients (13–17 years) is 0.5 mg once daily (Days 1–4), titrated to 1 mg (Days 5–7), and then to 2 mg on Day 8, based on clinical response and tolerability. Weekly increases of 1 mg are permitted, with a target dose of 2–4 mg daily and a maximum of 4 mg daily [53].

### QOI

Identify an appropriate dosing regimen for brexpiprazole in pediatric patients 13 years and older [52].

### Brexpiprazole pharmacometric model

The brexpiprazole PK profile is best described by a two-compartment model with sequential zero-first-order absorption and linear elimination. Modeling was done using NONMEM. Noncompartmental analysis was done in R. The derived PK metrics were: $\:AUC$, $\:{C}_{max}$, and $\:{C}_{trough}$.

Simulations confirmed that single-dose PK exposure in pediatric patients 13–17 years old at the proposed starting dose (0.5 mg) was 40% lower than in adults at the starting dose (1 mg), which was recommended for better tolerability in pediatric subjects. Additionally, steady-state PK exposure metrics ($\:AUC$, $\:{C}_{max}$, and $\:{C}_{trough}$), at 2 mg and 4 mg in pediatric patients 13–17 years old, were comparable to those in adults. Stratifying PK exposure metrics by body-weight quantiles revealed similar results between pediatric and adult patients, suggesting that weight-tiered dosing was unnecessary.

These results supported the extrapolation of brexpiprazole efficacy from adults to 13-17-year-old pediatric patients [52].

## Case study 5: mavacamten dosing regimen selection [54]

Mavacamten (CAMYZOS^®^), manufactured by MyoKardia and approved by the FDA in 2022, is a first-in-class cardiac myosin inhibitor approved for treating adults with symptomatic obstructive hypertrophic cardiomyopathy (HCM).

Mavacamten is metabolized by the cytochrome P450 2C19 (CYP2C19) enzyme, which is polymorphic. The frequencies of CYP2C19 poor metabolizers (those with two non-functional alleles, e.g., *CYP2C19* *2/*2, *2/*3, or *3/*3) are 2%, 4%, and 13% in those of European, African, and East Asian ancestry, respectively. The product package insert has a boxed warning for the risk of heart failure due to systolic dysfunction [55, 56]. Mavacamten half-life increases from 6 to 9 days in normal metabolizers to 23 days in CYP2C19 poor metabolizers. Mavacamten $\:AUC$ from time zero to infinity ($\:{AUC}_{0-\infty\:}$) is 241% greater in poor metabolizers, who have an increased risk of heart failure [55, 56]. These risks required evaluation of dose titration, therapeutic drug monitoring (TDM), and pharmacogenetic strategies in the drug development process.

In the Phase 3 study, mavacamten plasma concentration was measured using both TDM and echocardiography to guide individual dose adjustments. Due to the complexities of TDM, longer turnaround times for plasma concentration results, and frequent patient visits, a new echocardiography-guided dose-titration regimen was developed and evaluated.

### QOI

Does the clinical status and echocardiography-based dose-titration strategy provide safety and efficacy comparable to the combined TDM and echocardiography-based approach?

### Mavacamten pharmacometric model

Data from five different clinical studies were used for modeling and simulation. Using this dataset, E-R models were developed using nonlinear mixed-effects modeling to characterize both efficacy, via changes in Valsalva left ventricular outflow tract gradient (VLVOTg), and safety, via left ventricular ejection fraction (LVEF). The average concentration over the 168 h preceding the echocardiographic imaging ($\:{C}_{avg,\:168}$) was chosen as the exposure metric to reflect steady-state PK under weekly titration intervals. It was also highly correlated with observed trough concentrations.

The PK of mavacamten was described using a two-compartment model with first-order absorption. The E-R models, linked to the PopPK model, allowed simulations of individualized dose-E-R relationships in virtual patient cohorts, incorporating interindividual variability and CYP2C19 metabolizer phenotypes. Five echocardiography-guided regimens were simulated with this framework. The safety and efficacy profiles of Regimen #5 were comparable to those of the original TDM-based approach.

A model-informed drug development approach effectively captured the dynamics of the dose-titration regimen, demonstrating that the echocardiography-guided approach could replicate the safety and efficacy of the TDM plus echocardiography-guided regimen. CYP2C19 interaction simulations were conducted for different comorbidities. Based on the modeling, dose titration was combined with echocardiography-based efficacy (Valsalva left ventricular outflow tract gradient, VLVOTg) and safety (left ventricular ejection fraction, LVEF) monitoring was selected; TDM was not utilized. Mavacamten was approved for clinical use via a restricted Risk Evaluation and Mitigation Strategies (REMS) program [56].

## Case study 6: endpoint selection for tofersen [57]

Tofersen (QALSODY^®^) is an antisense oligonucleotide co-developed by Ionis Pharmaceuticals and Biogen that was approved by the FDA in 2023 to treat amyotrophic lateral sclerosis (ALS) associated with a superoxide dismutase-1 (*SOD1*) gene mutation.

The approved tofersen dosing regimen consists of three 100 mg intrathecally administered loading doses at 14-day intervals, followed by five 100 mg maintenance doses intrathecally administered every 28 days [58].

### QOI

The ALS Functional Rating Scale-Revised (ALSFRS-R), the validated rating instrument for monitoring the progression of disability in ALS and the primary endpoint in the pivotal VALOR trial of tofersen showed no statistically significant changes at Week 28 [59]. Evidence favoring tofersen treatment was found on several endpoints in the open-label extension phase at Week 52. As there is an urgent need for ALS treatments, plasma neurofilament light chain (NfL), a secondary endpoint, was identified as a surrogate biomarker because it is a biologically plausible biomarker linked to neuroaxonal injury and neurodegeneration, it is associated with ALS disease progression and was reduced in tofersen-treated patients. The reduction of NfL is nominally statistically significant at Week 28, but the NfL change was consistently observed for all subgroups based on sex, disease duration since symptom onset, and riluzole/edaravone use.

### Tofersen pharmacometric model

ANCOVA of ranked scores was used in the primary analysis to evaluate changes in ALSFRS-R at Week 28. Post hoc causal inference analysis was used to evaluate the mediatory role of plasma NfL reduction in tofersen’s effect on ALSFRS-R outcomes. By integrating data from multiple endpoints, including ALSFRS-R, survival, and NfL levels, the model highlighted the mechanistic relationship between plasma NfL reduction and slower disease progression. Patients with higher baseline NfL levels were shown to experience faster disease progression, reinforcing its prognostic value.

This analysis established plasma NfL as a reasonably likely surrogate endpoint, which, when combined with the totality of evidence, supported the accelerated approval of tofersen for SOD1-ALS, addressing an urgent need for this rare and life-threatening disease [57].

## Case study 7: patient selection for anakinra treatment [60]

Anakinra (KINERET^®^) is an interleukin-1 inhibitor manufactured by Swedish Orphan Biovitrum (Sobi) and is sold in partnership with Boehringer Ingelheim. Soluble urokinase plasminogen activator receptor (suPAR) is a predictive biomarker for progressive kidney injury that is elevated in moderate to severe coronavirus disease of 2019 (COVID-19). COVID-19 patients with suPAR ≥ 6 ng/ml were at high risk for requiring mechanical ventilation and mortality [61]. In the early days of the COVID-19 pandemic, the open-label SAVE trial and the randomized SAVE-MORE trials in Greece showed that suPAR-guided anakinra treatment was useful for preventing severe respiratory failure in COVID-19 patients [62, 63].

### QOI

Can individuals with suPAR levels of ≥ 6 ng/ml be identified from baseline characteristics, given that an approved suPAR assay was unavailable in the United States?

### Anakinra pharmacometric model

Machine learning methods were used to develop a scoring rule to identify patients likely to have elevated suPAR levels.

Two artificial intelligence/machine learning algorithms were used to predict individuals with suPAR level ≥ 6 ng/mL from their baseline characteristics. Elastic net regression identified key features. An artificial neural network was used to determine a cutoff value that maximized sensitivity while maintaining a positive predictive value of at least 0.95. Data from the SAVE-MORE trial was utilized to develop a scoring rule for identifying patients likely to have baseline suPAR levels ≥ 6 ng/ml, and the rule was externally validated using data from the SAVE trial [62, 63].

This marked the first time the FDA utilized AI/ML to identify the appropriate patient population for a clinical trial. The FDA approved anakinra treatment of COVID-19 under Emergency Use Authorization in 2022 [60].

## Case study 8: product approval for aripiprazole lauroxil [64]

Aripiprazole lauroxil (ARISTADA^®^) is a long-acting injectable form of the antipsychotic aripiprazole. It was approved by the FDA in 2015 for treating schizophrenia in adults and is marketed by Alkermes.

Several approved dosing regimens are currently available to meet individual patient’s needs, including 441 mg, 662 mg, or 882 mg administered monthly, 882 mg administered every 6 weeks, or 1064 mg administered every 2 months.

### QOI

The pivotal trials for aripiprazole lauroxil evaluated the 441 mg monthly, 882 mg monthly regimens [65]. Can aripiprazole lauroxil be safely and effectively dosed 662 mg monthly or 882 mg q6w?

### Aripiprazole pharmacometric model

A population-based PB-PK model was constructed in two stages. In the first stage, human PK data from several studies were used to develop an aripiprazole PBPK model. In the second stage, the aripiprazole model was extended to develop a PBPK model of aripiprazole lauroxil.

Aripiprazole is metabolized by CYP3A and CYP2D6 [64]. The aripiprazole lauroxil model accurately described the PK profiles, including the effects of CYP2D6 poor metabolizer (PM) status and the CYP3A inhibitor ketoconazole, and the CYP2D6 inhibitor quinidine, as well as the PK of aripiprazole after multiple oral doses.

The PK simulation and flat E-R relationship for the two studied 441 mg monthly vs. 882 mg monthly regimens enabled the approval of the new 662 mg monthly or 882 mg q6w regimens without additional trials.

## Case study 9: dose optimization for paliperidone palmitate long-acting injectable [66]

Paliperidone palmitate (INVEGA^®^) is also a long-acting injectable form of the atypical antipsychotic paliperidone, the primary active metabolite of risperidone, marketed by Johnson & Johnson. It was approved by the FDA in 2009 for acute and maintenance therapy of schizophrenia and schizoaffective disorder. Paliperidone palmitate is available as an oral extended-release (ER) formulation with a recommended maintenance dose of 6 mg q24 hours; doses of 12 mg q24 hours and 3 mg q24 hours are also approved for the ER formulation. Paliperidone palmitate doses are expressed as equivalents of paliperidone (eq) [67]. The sponsor developed an intramuscular long-acting injectable (LAI) form of paliperidone palmitate.

### QOI

Is the proposed regimen for the paliperidone palmitate LAI, consisting of two initial doses of 150 mg on Day 1, 100 mg on Day 8, and subsequent maintenance doses of 75 mg q4w (adjusted between 25 mg and 150 mg) starting Day 36, acceptable [68]?

### Paliperidone pharmacometric model

The proposed dosing regimens were evaluated using non-linear mixed-effects modeling with change in the positive and negative syndrome scale (PANSS) total score from baseline to the end of the treatment period as the primary efficacy endpoint. Simulations were conducted to obtain the predicted PK profiles for different dosing scenarios and used for dose optimization and regulatory evaluation.

Evaluation of the maintenance dosing regimen showed that the paliperidone exposure following the 150 mg initial dose and maintenance doses of 25 mg eq q4 weeks from the LAI is similar to the approved 12 mg q24 hours and 2 mg q24 hours dosing regimens of the ER oral formulation.

The proposed paliperidone palmitate long-acting injectable form initial dose of 150 mg eq on Day 1 and 100 mg eq on Day 8 was evaluated in two scenarios: for patients who had been receiving either paliperidone or a different antipsychotic treatment. In both scenarios, the proposed initial dosing regimen achieved the desirable exposure within one week, and the peak exposure was in the concentration range found to be safe and well tolerated.

The dosing windows of the second initial dose and maintenance doses of paliperidone palmitate long-acting injectable form were also evaluated. Paliperidone exposure levels remained similar if the second initial dose was administered within 2 days and the maintenance doses were administered within 1 week of the scheduled time.

Three missed dose scenarios were evaluated in simulations. Patients who missed doses for more than 6 months are restarted again with the initial doses of 150 mg on Day 1, 100 mg on Day 8, and maintenance dosing of 75 mg eq (adjusted between 25 mg and 150 mg) q4 weeks. Patients who missed treatment for 6 weeks to 6 months were restarted with initial doses on Day 1 (100 mg maximum) and 100 mg on Day 8, and subsequent 75 mg eq (adjusted between 25 mg −150 mg) q4w maintenance doses. Resumption of the regular q4w maintenance dose was recommended for patients who missed dosing for a duration of one month to 6 weeks.

## Future directions, opportunities, and challenges

### Future directions

MIDD is a powerful tool for supporting new drug development and regulatory decision-making [69]. The scope of its applications has been expanding. Concomitantly, the availability of new therapeutic modalities such as silencing RNA (siRNA), new variants of antibodies and antibody-drug conjugates, gene therapies, chimeric antigen receptor (CAR) T cells, and other cell-based therapies has proliferated. Yuan et al. have reviewed the utility of MIDD for siRNA drugs [70]. Belov et al. review the application of MIDD to address knowledge gaps in CAR T cell therapies and adeno-associated virus gene therapies [71].

The FDA has issued a roadmap for reducing animal testing in preclinical safety studies with new approaches such as microdosing and imaging in human volunteers, ex vivo human tissues, in vitro human-based systems, and in silico modeling approaches, including PB-PK, AI/ML, and QST/QSP [72]. The roadmap has an initial focus on monoclonal antibody testing. This may be an important future direction for MIDD efforts based on our case studies, which highlighted the impact of MIDD on the development of monoclonal antibody products.

AI methods, such as deep learning, generative AI, and large language models, are starting to transform drug discovery pipelines, drug development, and pharmacometrics [73–79]. The anakinra case study demonstrates the utilization of AI methods for patient selection in an MIDD framework [60]. AI methods enable the integration of large external data sources into MIDD [75, 76, 80]. The FDA has issued guidance for the use of AI/ML to support regulatory decision-making that incorporates COU, model risk, and credibility assessment [81].

The FDA also launched a new LLM-based tool, ELSA, in June 2025 to accelerate scientific evaluations and clinical protocol reviews [82]. ELSA operates in a closed government cloud environment to avoid exposure of the sponsor’s documents containing intellectual property on the internet. The utilization of LLM-based tools by the FDA in their MIDD workflows may foster the use of LLM-based software tools by sponsors to conduct self-assessments of their regulatory materials.

### Opportunities

The mission of ICH M15 is to achieve consistency of MIDD practices and implementations. However, regulatory agencies, even divisions within each agency, can vary in their confidence in modeling and simulation results and have different levels of risk tolerance in decision-making. As with any emerging yet transformative process, variations in MIDD documentation standards and assessment expectations are likely as regulatory agencies evaluate the safety of medical products for their respective populations and environments. Harnessing the full benefits of MIDD harmonization requires ongoing dialogue and a common understanding among global regulators themselves and between regulatory agencies and globally active pharmaceutical industry representatives [83].

One of the main goals of the ICH M15 guideline is to provide a common framework for the content and organization of model-based analyses in regulatory submissions. Although the acceptability of model-based analyses to support regulatory decisions currently varies across global regulatory agencies, the implementation of the ICH M15 guideline is expected to lead to greater consensus in the future. The M15 guideline also provides a harmonized risk-based framework for determining the level of rigor required for an analysis, which varies depending on the impact of the analysis on regulatory decision-making. It is anticipated that there will be increasing convergence on the acceptability of model-based analyses both across and within regulatory agencies to inform regulatory decisions.

MIDD is now an integral component of clinical development in many pharmaceutical companies. The ICH M15 guideline and successful track record of MIDD in regulatory submissions provide greater clarity to executive leadership on the appropriate use of MIDD to support clinical development goals. As a result, experienced modeling scientists are now routinely included in the clinical development planning process to develop and implement appropriate modeling strategies to address drug development questions of interest. Successful MIDD case studies are real-world exemplars of the strategic use of modeling and simulation methodologies in regulatory interactions. They can be used to design bridging studies, select pharmacometric modeling methods, and PK endpoints for drug product development. Likewise, early career scientists involved in the data acquisition, study design, and model-based analyses can also benefit from gaining an understanding of the effective use of MIDD.

Bi et al. review how the E-R relationship from MIDD can be used to select “exposure-matched” dosing regimens for pediatric populations based on available information from adult trials; PB-PK modeling has also been bridged with ontogeny data to predict PK in neonates and infants [84, 85]. Yang et al. have reviewed the use of PB-PK models for virtual populations across the lifespan, as well as for renal and hepatic impairment [86].

The adalimumab case study of hidradenitis suppurativa (Supplementary File) exemplifies the use of MIDD to expand treatments for a rare disease [44]. The recruitment and clinical trial design challenges in rare diseases present opportunities for the MIDD approach to shine [87].

There have been ongoing technological advances in dosage form options for drug development. Most notably, there have been several successes with long-acting injectable dosage forms that can mitigate patient adherence issues in challenging diseases such as schizophrenia and opioid use disorders. Long-acting injectables use diverse biopharmaceutical strategies to achieve duration of action. For schizophrenia, intramuscular Invega Sustenna^®^, an extended-release suspension of paliperidone (or 9-hydroxyrisperidone) palmitate, subcutaneous Perseris™, which is based on Atrigel technology [88] and forms a solid implant on in vivo injection, and intramuscular Risperdal Consta, a suspension dosage form of risperidone in polylactide coglycolide microspheres, are all LAI formulations of the second-generation antipsychotic drug risperidone; long-acting depot formulations of olanzapine pamoate are formulated as a suspension and salt form dissolves slowly over time and dissociates into the free base form of olanzapine. An extended-release formulation of buprenorphine that is administered once monthly was approved in 2017 for treating opioid-use disorder. The aripiprazole lauroxil case study exemplifies using MIDD for a long-acting injectable dosage form [64]. The MIDD considerations for long-acting injectables are reviewed by Sharan et al. [89].; the use of MIDD for other long-acting injectables in clinical scenarios such as contraception and malaria therapeutics has also been reviewed [90, 91].

### Challenges

This review, along with the case studies, underscores the broad range of impacts that MIDD activities have on drug development and regulatory processes. The examples illustrate the usefulness of the dose-exposure relationships from MIDD for dose extrapolation to related disease indications, dosage forms, and clinical populations without the need for additional clinical trials. However, MIDD, in its current form, requires access to a sufficient body of clinical evidence for the same drug in the relevant clinical population to facilitate model building.

The use of PB-PK models in drug development and regulatory submissions has continued to expand. However, a review of EMA marketing authorization applications submitted in 2022–2023 found that most of the PB-PK models were not considered qualified for the intended use due to issues with model structure, the lack of relevant data for model validation, poor prediction of clinical data, and weak justification for model assumptions and parameters [12]. Likewise, the availability of reliable, robust, and generalizable pharmacometric models is the cornerstone for any MIDD effort in drug product development. It is recognized that such “model instability” deficiencies can emerge in pharmacometric model performance in use-reuse, repurposing, and new data settings. In particular, modeling drug absorption from alternative routes of administration and dosage forms can be more challenging to model [92]. The lack of robustness and generalizability can result from insufficient information in the training data to support parameter estimation or due to overly complex models. Workflows to test and address these issues are emerging [93].

It should be acknowledged that aspects of the MIDD process represent a paradigm shift from the traditional study design and statistical methods preferred by clinical investigators and statisticians involved in drug trials, who may be unfamiliar with drug development considerations, PK, and E-R relationships, pharmacometric modeling assumptions, and techniques. The clarity of MIDD terminology, the pre-specification of assessment methods, and the growing track record of MIDD successes across therapeutic classes will likely promote wider acceptance.

Notably, the MIDD framework has enabled the approval of drugs for indications that might have been delayed or never pursued under conventional regulatory standards. In the case of nivolumab and atezolizumab, MIDD made the approval of dosing regimens for multiple cancers and indications feasible, thereby benefiting patients. MIDD has enabled accelerated, even simultaneous approvals of drugs for pediatric conditions. In rare disease settings, MIDD can be indispensable because it is impossible to recruit enough patients for efficacy studies.

Just as MIDD was adapted from engineering disciplines, the successes and lessons learned from utilizing MIDD in drug development and regulatory agencies could similarly promote the adoption of these approaches in fields such as personalized medicine, clinical trial design, healthcare, and public health.

## Supplementary Information

Supplementary Material 1.

## Data Availability

No datasets were generated or analysed during the current study.

## Declarations

### Use of artificial intelligence

Artificial intelligence-enabled tools, such as Grammarly, Microsoft Word, and ChatGPT, were used to improve readability and language. Bibliographic tools, including Google and EndNote, helped curate references, add citations, and generate bibliographies. The generative AI features of graphics tools, like Adobe Illustrator, were utilized to create prototypes for some figures.
