# Supplementary material for: Scoping review of the role of pharmacometrics in model-informed drug development
Source: J Pharmacokinet Pharmacodyn. 2025 Oct 15;52(6):56. doi: 10.1007/s10928-025-10005-8 (PMC12528282; doi:10.1007/s10928-025-10005-8)
Supplement: Supplementary file 1 — Supplementary Material 1. [file 10928_2025_10005_MOESM1_ESM.pdf]

# SUPPLEMENTARY FILE

# Scoping Review of the Role of Pharmacometrics in Model-Informed Drug Development

Amruta Gajanan Bhat [1], Euibeom Shin [1], Amit Roy [2], and Murali Ramanathan [1]

[1] Artificial Intelligence and Pharmacometrics Laboratory, Department of Pharmaceutical Sciences. University at Buffalo, The State University of New York, Buffalo, NY, USA.

[2] PumasAI, Dover, DE, USA.

**CORRESPONDING AUTHOR:** Murali Ramanathan
355 Pharmacy, Department of Pharmaceutical Sciences
University at Buffalo, Buffalo, NY 14214-8033.
Phone: (716)-645-4846. E-mail: Murali@Buffalo.Edu

**Running Head**: Pharmacometrics and MIDD
**Keywords**: MIDD, Pharmacometrics, AI, Study Design

## SUPPLEMENTARY CASE STUDIES

### **Case Study S1: Change of Route of Administration for Secukinumab** (1)

**Secukinumab:** Secukinumab (COSENTYX®) is a fully human monoclonal antibody targeting interleukin-17 (IL-17) marketed by Novartis. Secukinumab via the subcutaneous route of administration was approved to treat ankylosing spondylitis (AS) and psoriatic arthritis (PsA) in 2016, and non-radiographic axial spondylarthritis (nr-axSpA) in 2020 (1).

The dosing regimen with a loading dose is 150 mg at Weeks 0, 1, 2, 3, and 4 and every 4 weeks (q4w) thereafter. Without a loading dose, the regimen is 150 mg q4w, and if a patient with PsA or AS continues to have active disease, 300 mg q4w can be considered.

Novartis sought the marketing registration for an intravenous (IV) formulation of secukinumab as part of its product lifecycle management process. They conducted two Phase III studies to support the secukinumab IV formulation. Concerns about the higher exposure and limited 16-week safety data led to an MIDD approach to identify an appropriate IV dosing regimen to evaluate long-term safety.

**QOI:** What is the IV dose of secukinumab for treating psoriatic arthritis and ankylosing spondylitis?

**Secukinumab Pharmacometric Model:** A two-compartment disposition model with first-order elimination and first-order absorption for the SC administration and constant rate infusion for the IV administration was used to model secukinumab PK.

Goodness-of-fit diagnostic plots (internal validation) were used to assess whether the model adequately captured the data trends. Data from independent clinical studies (external validation) were used to ensure the model's predictive accuracy.

The FDA independently assessed and verified the PopPK model by comparing observed and predicted values for key PK parameters.

The PK bridging technique to comparing IV vs. SC safety and efficacy data provided a robust benefit-risk assessment and supported the IV approval without additional clinical trials.

**Case Study S2: Decreasing the Dosing Frequency for Cetuximab** (2)

**Cetuximab:** Cetuximab (ERBITUX®)is an epidermal growth factor receptor (EGFR) inhibitor manufactured by Eli Lilly. It was approved by the FDA in 2004 for metastatic, KRAS wild-type colorectal cancer and squamous cell head and neck cancers (SCCHN) (3)

The approved dosing regimen consists of an initial dose of 400 mg/m$^2$ followed by 250 mg/m$^2$ weekly. Real-world evidence showed no significant difference in efficacy between the q1w and q2w regimens, and the q1w regimen benefited patients while improving resource utilization.

**QOI:** Is a 500 $mg/m^2$ q2w dosing regimen as effective and safe as the approved q1w 250 $mg/m^2$dosing?

**Cetuximab Pharmacometric Model:** A two-compartment model with saturable elimination was developed to compare the cetuximab PK of q1w and q2w regimens. MIDD simulations of PK in both dosing regimens showing the overlap in exposure measures in both mCRC and SCCHN patients, and showing the clinical efficacy enabled the approval of the cetuximab without additional clinical trials. The PopPK simulations, with concentration-time profiles overlapping, were validated against known PK parameters. An incidence rate meta-analysis was conducted. Survival outcomes were analyzed between the q2w and q1w cohorts using propensity score matching. The FDA independently assessed steady-state drug concentrations ($C_{avg,ss}$ $C_{min,ss}$, $C_{max,ss}$) for both dosing regimens, which was further supported by clinical safety and efficacy data.

**Case Study S3: Shortening the Infusion Duration for Ramucirumab** (2)

**Ramucirumab:** Ramucirumab (CYRAMZA®) is a monoclonal antibody targeting vascular endothelial growth factor receptor 2 (VEGFR-2) marketed by Eli Lilly. It is used in combination with docetaxel to treat metastatic non-small cell lung cancer (NSCLC). It is used as a single agent or in combination with paclitaxel for advanced gastric cancer. The FDA approved its use in 2014.

The ramucirumab dosing regimen for advanced gastric junction adenocarcinoma is 8 mg/kg every 2 weeks. For NSCLC, the recommended dose of ramucirumab is 10 mg/kg, administered intravenously on day 1 of a 21-day cycle. The infusion duration for all indications is 60 minutes. Patients receiving ramicirumab are pre-medicated with an IV histamine antagonist (e.g., diphenhydramine hydrochloride) to mitigate the risk of infusion-related reactions (IRR). Patients with prior IRR are also premedicated with dexamethasone.

**QOI**: Based on real-world evidence, a 30-minute ramucirumab infusion was proposed, which reduces the time burden for patients and medical staff. Does the 30-minute infusion rate have a greater safety risk due to IRR?

**Ramucirumab Pharmacometric Model:** A two-compartment model with saturable elimination was used to compare ramucirumab PK after administering the 30-minute and 60-minute infusions.

MIDD evidence showed no meaningful differences in PK or safety outcomes between the 30-minute and 60-minute infusion. The incidence of IRRs across different infusion rate quartiles was comparable, and no increased risks were predicted. These findings were combined with clinical and scientific data to justify reducing the ramucirumab infusion time from 60 minutes to 30 minutes.

**Case Study S4: Adalimumab in Adolescents with Hidradenitis Suppurativa** (4)

Adalimumab (HUMIRA®), marketed by AbbVie, is a monoclonal antibody that targets human tumor necrosis factor-alpha (TNF-α) (5). In 2015, Adalimumab received FDA approval to treat various autoimmune conditions such as rheumatoid arthritis, AS, PsA, Crohn's disease, and ulcerative colitis (4), and moderate-to-severe hidradenitis suppurativa (HS, a chronic, inflammatory skin condition that causes skin abscesses) (6) in adults.

The approved dosing regimen was 160 mg on Day 1 and 80 mg on Day 15, followed by maintenance dosing of 40 mg every week starting Day 29.

AbbVie proposed a MIDD approach to support dose selection and regulatory evaluation of an adalimumab dosing regimen in adolescent HS patients due to the absence of clinical PK data and efficacy trials for this population.

**QOI:** Is it possible to expand the adult adalimumab dosing regimen for adolescent HS patients aged 12 years and older, weighing 30 kg or more?

**Adalimumab Pharmacometric Model:** A linear one-compartment model with first order absorption and elimination and a combined proportional and additive residual error model was developed to model the PK of adalimumab.

Simulations based on popPK models performed on various dosing regimens in adolescent HS patients using exposure metrics such as time-averaged concentration ($C_{avg,ss}$), trough

concentration ($C_{trough,ss}$), and peak concentration at steady state ($C_{max,ss}$) compared to those in adult patients enabled the approval of the adalimumab with safety and efficacy data without additional clinical trials.

Modeling was used to predict exposure-response and assess the safety and efficacy profile of weight-tiered dosing regimens for adolescent patients. The model outcome was compared against observed PK data from 521 pediatric and 3,095 adult patients across 17 clinical studies by simulating various dosing regimens and comparing predicted concentrations to observed data in adults and pediatric populations.

The model's fit was evaluated using graphical and numerical methods. The predicted PK metrics ($C_{avg,ss}$ $C_{trough,ss}$, $C_{max,ss}$) were compared with existing data for pediatrics and adult populations across weight ranges and dosing regimens.

## REFERENCES

(1) Pisal, D.S. *et al*. Model-Informed Drug Development-Based Approval of Intravenous Secukinumab for the Treatment of Adult Patients with Active Psoriatic Arthritis, Active Ankylosing Spondylitis, and Active Non-Radiographic Axial Spondyloarthritis. *Clin Pharmacol Ther*, (2024).

(2) Ni, L. *et al*. Optimizing the Dosing Regimen of Cetuximab and Ramucirumab Using the Model-Informed Drug Development Paradigm. *Clin Pharmacol Ther* **114**, 77-87 (2023).

(3) Chidharla, A., Parsi, M. & Kasi, A. Cetuximab. In: *StatPearls* (Treasure Island (FL), 2025).

(4) Bi, Y. *et al*. Model-Informed Drug Development Approach Supporting Approval of Adalimumab (HUMIRA) in Adolescent Patients with Hidradenitis Suppurativa: a Regulatory Perspective. *AAPS J* **21**, 91 (2019).

(5) Ellis, C.R. & Azmat, C.E. Adalimumab. In: *StatPearls* (Treasure Island (FL), 2025).

(6) Ballard, K. & Shuman, V.L. Hidradenitis Suppurativa. In: *StatPearls* (Treasure Island (FL), 2025).
